# The concept and definition of therapeutic inertia in hypertension in primary care: a qualitative systematic review

**DOI:** 10.1186/1471-2296-15-130

**Published:** 2014-07-02

**Authors:** Jean-Pierre Lebeau, Jean-Sébastien Cadwallader, Isabelle Aubin-Auger, Alain Mercier, Thomas Pasquet, Emmanuel Rusch, Kristin Hendrickx, Etienne Vermeire

**Affiliations:** 1 Department of General Practice, EES, University of Tours, 10 Boulevard Tonnellé, BP 3223, 37032 Tours, Cedex 1, France; 2 Department of General Practice, University Paris Diderot, Sorbonne Paris Cité, France; 3 Department of General Practice, University of Rouen, Rouen, France; 4 Department of Public Health, EES, University of Tours, Tours, France; 5 Department of Primary and Interdisciplinary Care, University of Antwerp, Antwerp, Belgium; 6 Department of Nursing and Midwifery, University of Antwerp, Antwerp, Belgium

**Keywords:** Hypertension, Primary care, Quality of health care, Systematic review, Therapeutic inertia

## Abstract

**Background:**

Therapeutic inertia has been defined as the failure of health-care provider to initiate or intensify therapy when therapeutic goals are not reached. It is regarded as a major cause of uncontrolled hypertension. The exploration of its causes and the interventions to reduce it are plagued by unclear conceptualizations and hypothesized mechanisms. We therefore systematically searched the literature for definitions and discussions on the concept of therapeutic inertia in hypertension in primary care, to try and form an operational definition.

**Methods:**

A systematic review of all types of publications related to clinical inertia in hypertension was performed. Medline, EMbase, PsycInfo, the Cochrane library and databases, BDSP, CRD and NGC were searched from the start of their databases to June 2013. Articles were selected independently by two authors on the basis of their conceptual content, without other eligibility criteria or formal quality appraisal. Qualitative data were extracted independently by two teams of authors. Data were analyzed using a constant comparative qualitative method.

**Results:**

The final selection included 89 articles. 112 codes were grouped in 4 categories: terms and definitions (semantics), “who” (physician, patient or system), “how and why” (mechanisms and reasons), and “appropriateness”. Regarding each of these categories, a number of contradictory assertions were found, most of them relying on little or no empirical data. Overall, the limits of what should be considered as inertia were not clear. A number of authors insisted that what was considered deleterious inertia might in fact be appropriate care, depending on the situation.

**Conclusions:**

Our data analysis revealed a major lack of conceptualization of therapeutic inertia in hypertension and important discrepancies regarding its possible causes, mechanisms and outcomes. The concept should be split in two parts: appropriate inaction and inappropriate inertia. The development of consensual and operational definitions relying on empirical data and the exploration of the intimate mechanisms that underlie these behaviors are now needed.

## Background

### The burden of uncontrolled hypertension

The burden of hypertension weighs heavily on public health in all industrialized countries [1]. Surveys in Europe and North America show prevalences of 40–80% in patients aged 35–64 years [2]. Hypertension leads to a major risk of stroke and acute myocardial infarction (AMI), with morbidity and mortality increasing linearly with increases in both systolic and diastolic blood pressure (BP) [3]. At the same time, there is strong evidence for the major benefits of treating hypertensive patients, resulting in a reduced risk of up to 15% for AMI, 40% for stroke, and 30% for cardiovascular mortality [4].

Considering this evidence, a number of guidelines have been published on the diagnosis, treatment, and follow-up of hypertensive adult patients, either by health authorities or by scientific colleges and societies [5-7]. The way drugs should be used and combined, the treatment targets, and elements of the patients’ education and follow-up have been clearly and thoroughly expressed in these guidelines.

Despite this clearly codified and evidence-based assessment, real-life primary care shows quite disappointing results. The classical rule of halves still holds in most European countries: approximately half of the patients with a diagnosis of hypertension are not treated and half of those who are treated do not reach set targets [8]. Although the situation is somewhat better in the US, where two-thirds of the diagnosed patients reach the therapeutic goals, there is still room for improvement [9].

A number of reasons can be put forward to explain these poor results: many are related to the health system and to the patients (notably adherence to treatment). Other reasons are related to health-care providers, and particularly to clinical inertia.

### Clinical and therapeutic inertia

Clinical inertia was initially defined in 2001 by Phillips [10]. According to this definition, clinical inertia applies only to the management of risk factors, when therapeutic targets are clearly defined and the benefits to reach those targets are well established. Effective therapies should be widely available, and practice guidelines disseminated extensively. Clinical inertia appears whenever the health-care provider does not initiate or intensify therapy appropriately when therapeutic goals are not reached: “recognition of the problem, but failure to act”. Phillips described three main sets of reasons for therapeutic inertia: overestimation of care, soft reasons (i.e. “improving control”, “target almost reached”, etc.), and lack of training and organization in the practice at “treating to target”. Subsequent articles added clinical uncertainty [11] and competing demands [12] as other reasons for clinical inertia. This initial definition was Phillips’ own idea, and was produced on a deductive basis. With the exact same definition, Okonufa *et al*. introduced the terms “therapeutic inertia” in 2006 [13]. Since then, the terms “clinical inertia” and “therapeutic inertia” have been used indistinctly (we chose to use the latter in this article). Neither of them, nor “inertia” alone, is a Medical Subject Heading (MeSH) term.

Clinical inertia as defined by Phillips has become increasingly acknowledged as a major impediment to reaching both individual and public-health targets for a number of risk factors [13]. Hypertensive patients, in particular, experience therapeutic inertia from their physician in up to 85% of visits in some European countries [9]. On the other hand, Phillips *et al.* themselves gave a note of caution in their paper that exceptions occur and that appropriate care should allow individualization: “*the uniform application of guidelines for patient management could result in overtreatment or inappropriate action*” [10].

Since then, this major ambivalence nested in the core of the concept has plagued all research on mechanisms and outcomes of clinical or therapeutic inertia, and all experimental attempts to reduce it. Very few studies have tried to clarify the concept or to refine the definition of therapeutic inertia from empirical data, to make it operational on an inductive basis.

We have conducted a systematic review of the literature on therapeutic inertia in hypertension, and have looked for elements of its definition and conceptualization. Our aim was to come up with a clear concept and to form an operational definition upon which clinical trials could rely.

## Methods

As much as possible, we have tried to report this review according to the PRISMA guidelines [14]. However, these guidelines were designed for the report of quantitative systematic reviews and meta-analysis, and a number of item could not be considered here.

### Types of studies considered for the review

Because we were looking for definitions of a recent concept, we considered that every type of paper could be eligible:

– Trials

– Surveys and epidemiological studies

– Qualitative research

– Reviews

– Opinion papers and editorials about the concept of inertia or about guideline-implementation issues

### Search strategy for identification of studies

#### *Databases*

The following databases were searched from their beginnings until June 2013: Medline, EMbase, PsycInfo, the Cochrane library and databases, BDSP (French Public Health Database), CRD (Center for Reviews and Dissemination), and NGC (National Guideline Clearinghouse).

The search algorithm for Medline (via PubMed) was: (“guideline adherence”[MeSH Terms] OR (“practice guidelines as topic”[MeSH Terms] AND (“clinical audit”[MeSH Terms] OR “clinical competence”[MeSH Terms] OR “attitude of health personnel”[MeSH Terms] OR “delivery of health care”[MeSH Terms] OR “physician’s practice patterns”[MeSH Terms] OR “nurse’s practice patterns”[MeSH Terms])) AND (“hypertension”[MeSH Terms] OR “antihypertensive agents”[MeSH Terms])) OR “clinical inertia”[All Fields] OR “therapeutic inertia”[All Fields]. The other databases were searched using the same algorithm adapted to their respective syntactic structures.

Languages were limited to English, French, Spanish, Portuguese, German, and Dutch.

#### *Additional searches*

Google Scholar was systematically searched for “clinical inertia” and “therapeutic inertia”. All results retrieved by searching Google with the same terms were explored. The reference list of each selected article was systematically screened for other relevant articles. Experts in the field were contacted and asked for their personal databases.

### Methods of the review

#### *Abstracts selection*

Two researchers (JPL and TP) reviewed independently the titles, abstract sections, and keywords of every record retrieved, using a score list. The article was included if one of the following characteristics was present:

– The words “clinical inertia” or “therapeutic inertia” appeared

– Hypertension guidelines implementation was the main subject

– Design, assessment, or evaluation of any kind of intervention directed to the general practitioner for hypertension control was the main topic

– The general practitioners’ behaviors or barriers to change regarding hypertension treatment were the main topic.

Although these last topics were not part of the research question, chances were that the concept of therapeutic inertia would be discussed in such articles.

#### *Full-texts assessment*

The articles were rejected if they recorded no element of definition or conceptualization. Articles which only cited the words “therapeutic inertia” or “clinical inertia” with no further explanation, or which referred directly and explicitly to the initial publication by Phillips without any restriction or discussion about its content were rejected. Epidemiological surveys that measured the gap between actual care for hypertension and guidelines but did not discuss the mechanisms of poor implementation were also rejected, as they did not provide any criteria or element of a definition for therapeutic inertia.

Agreement between the researchers was calculated using Cohen’s kappa. Differences in opinion were resolved by discussion that included a third researcher (JSC).

#### *Process of data collection*

A constant comparative qualitative method according to grounded theory was used to collect and classify emerging data from the full articles [15,16]. Units of text (words, sentences, paragraphs) were labeled through an open-coding process. A lexical analysis was carried out simultaneously, which focused on the words used to comment on inertia. Axial coding was then conducted, which consisted of comparing and grouping codes together into categories. Finally, through a selective coding process, all the categories were organized hierarchically according to their reliability and consistency, which led to an accurate description of the emerging concepts.

Data were independently analyzed from each article by the two teams of researchers (JPL/TP and IAA/AM), using a qualitative analytical software package (NVivo 9.2, QSR International Pty Ltd, Doncaster, Australia; 2011). Discrepancies were resolved by discussion, and any disagreement went to arbitration with a fifth researcher (JSC).

## Results

### Search results

**Figure 1:**
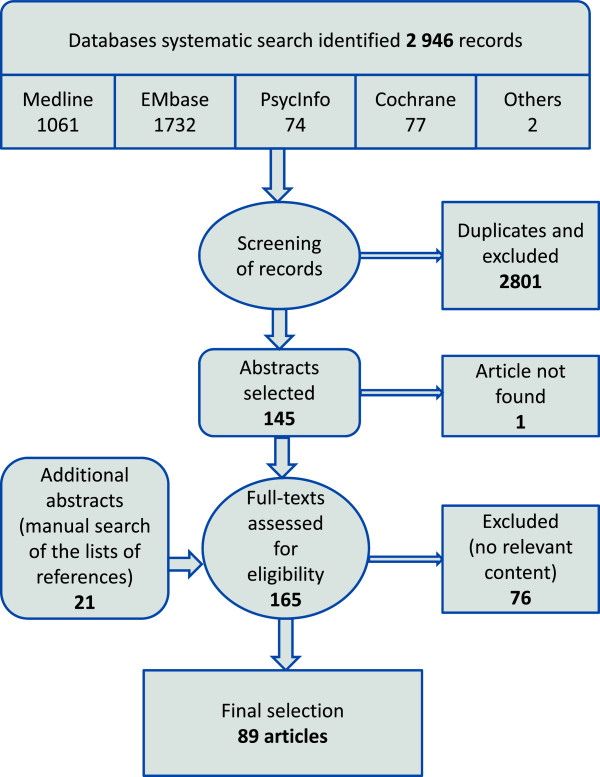
Systematic research flow chart.

The initial search of the databases resulted in a total of 2 946 abstracts: 1061 from Medline, 1732 from EMbase, 74 from PsycInfo, 77 from the Cochrane Library and database, and 2 from the other databases. After reviewing the abstracts, removing duplicates, and discussion to resolve differences in opinions, 145 abstracts were included: 84 from Medline, 51 from EMbase, 8 from PsycInfo, 2 from the Cochrane Library and database, and none from the other databases (duplicates were removed in this order). Systematic check of all the results retrieved in Google with the terms “clinical inertia” (162 results) and “therapeutic inertia” (142 results) did not lead to any new inclusion, and neither did the personal databases of the authors and the experts we contacted. A systematic search of the reference lists of selected articles retrieved 21 more abstracts. Inter-reviewer agreement at this stage, expressed as the observed Cohen’s kappa was 0.84 (95% CI: 0.80–0.88).The full-texts of 165 articles were assessed and checked for relevant content independently by two researchers (JPL and TP): 76 did not contain any relevant information, and 1 article could not be found (no archive kept by the journal nor by the author). The final selection included 89 articles (Figure 1).

### Types of publications and their contents

Of the 89 articles, 36 were clinical studies (8 trials, 16 cross-sectional studies, 7 cohort studies and 5 surveys), 5 were qualitative studies (including a nominal group consensus study), 15 were literature reviews – of which none matched the criteria of a systematic review -, 10 were editorials, and the other were experts opinion or position articles (including commentaries and 2 letters) (see Additional file 1).

### Coding

Open coding of the relevant content of the articles resulted in 112 codes. These open codes were grouped for analyses into four categories: “terms and definitions” (semantics), “who” (physician, patient or system), “how and why” (mechanisms and reasons), and “appropriateness”.

#### *Terms and definitions*

Most authors considered that “clinical inertia”, “therapeutic inertia” and “physician inertia” were all synonymous. This was either explicit [17,18] or implicit when Phillips’ definition of clinical inertia was quoted to define therapeutic inertia [11] or physician inertia [19]. A few authors, however, insisted that different terms should mean different things: “*the terms “clinical inertia” and “therapeutic inertia” have been used recently by authors, primarily to attribute to physicians the apparent failure of patients to attain therapeutic blood pressure goals. We think it would be helpful to define and differentiate these terms*” [20]. Scheen made a clear distinction, stating that: “*Therapeutic inertia is one of the components of clinical inertia*”, but gave no further explanation [21]. Giugliano *et al*. made a distinction between the overall phenomenon of clinical inertia and the part that was attributable to the physician’s behavior: i.e., physician inertia [22], a term also used by Krakoff *et al.*, but without any distinction from clinical inertia [19]. Gil-Guillén *et al*. separated “diagnostic inertia” from “therapeutic inertia” : “*Diagnostic inertia was identified when a patient without known hypertension had high blood pressure (BP) but was labeled “normal” by the medical staff, and therapeutic inertia when treatment was not modified for a hypertensive patient on the presence of high BP values*” [23]. Vinyoles proposed three kinds of inertia: “*Three inertias are barriers to change: physician’s inertia, patient’s inertia, and health authorities inertia*”, but the respective definitions remained implicit [24]. Discussing the synergy between patient non adherence and healthcare provider inertia, Reach proposed “Clinical myopia” for the common mechanism underlying these behaviors [25].

While the presumed causes of clinical inertia were widely discussed in a number of articles, considerations on the definition remained scarce. A few authors pointed out that there was a need for an accurate definition: “*While there is additional history behind the use of the terms “clinical inertia” and “therapeutic inertia,” much of the more recent usage is imprecise. We think that it is time to use these terms more carefully and more purposefully and to refer to models that have some basis in theory and evidence*” [20].

A few specific elements were not clear about Phillips’ definition. Ardery *et al*. considered that: “*Infrequent documentation of lifestyle recommendations could reflect another type of clinical inertia—namely, missed opportunities to promote patient self-management*” [26]. Gugliano *et al.* stated that: “*Clinical inertia also may apply to the failure of physicians to stop or reduce therapy no longer needed*” [22], a situation for which Rodrigo *et al.* proposed the specific term “therapeutic momentum” [27], although this term had already been defined as synonymous to clinical inertia by Faria *et al*. [17].

Scheen considered that the actual term “inertia” already meant “unjustified”: “*therapeutic inertia can be defined as an unjustified delay in treatment initiation or intensification*”, or “deleterious”: “*a caregiver behavior resulting in a deleterious delay*” [21]. The possible occurrence of a justified or beneficial delay was not discussed in this article. Looking for an operational definition, O’Connor concluded that: “*Flexibility in how clinical inertia is defined could be seen by some as a limitation. However, from the point of view of care improvement, this sort of flexibility may often be an advantage because it allows local tailoring of initiative and interventions.*” [28]. The terms retrieved and their initial or modified definitions are listed in Table 1.

**Table 1:** Terms and definitions

**Term**	**First occurrence**	**Definition**
Clinical inertia	Phillips *et al*.. *Ann Intern Med* 2001,**135**:825–834.	*Health care providers often do not initiate or intensify therapy appropriately during visits of patients with these problems* [hypertension, dyslipidemia and diabetes]*. We define such behavior as clinical inertia—recognition of the problem, but failure to act.*
Therapeutic inertia	Okonufa *et al*.. *Hypertension* 2006,**3**:345–351.	*Therapeutic inertia (TI), that is, failure of providers to begin new medications or increase dosages of existing medications when an abnormal clinical parameter is recorded.*
Patient's inertia	Vinyoles. *Hipertension* 2007,**24**:91–92.	*Three inertias are barriers to change: physician's inertia, patient's inertia, and health authorities inertia.*
		(Translated from Spanish)
Health authorities inertia	Vinyoles. *Hipertension* 2007,**24**:91–92.	*Three inertias are barriers to change: physician's inertia, patient's inertia, and health authorities inertia.*
		(Translated from Spanish)
Physician inertia	Vinyoles. *Hipertension* 2007,**24**:91–92.	*Three inertias are barriers to change: physician's inertia, patient's inertia, and health authorities inertia.*
		(Translated from Spanish)
	Moser *et al*.. *J Clin Hypertens* 2009,**11**:1–4.	*Physician inertia is defined as the failure to initiate therapy or to intensify or change therapy in patients with BP values >140 ⁄90 mmHg, or >130⁄80 mm Hg in hypertensive patients with diabetes, renal, or coronary heart disease.*
Clinical Myopia	Reach. *Diabetes Metab* 2008,**34**:382–385.	*We suggest that a failure to give preference to the long-term benefits of treatment intensification may represent a common mechanism underlying both patient non-adherence and physician clinical inertia. We dub such a failure as “clinical myopia”.*
Therapeutic momentum	Faria *et al*.. *J Am Soc Hypertens* 2009,**3**:267–276.	*Therapeutic inertia, therapeutic momentum, and physician inertia are all terms synonymous with clinical inertia*
	Rodrigo *et al*.. *Int J Clin Pract* 2013,**67**:97–98.	*The reluctance to step down or withdraw therapy when further prescription is not needed or not supported by evidence. We have termed it ‘therapeutic momentum’.*
Diagnostic inertia	Gil-Guillén *et al*.. *Blood Press* 2010,**19**:3–10.	*Diagnostic inertia was defined as a failure to consider the diagnosis of HTN in a subject in the absence of diagnosis of HTN and elevated BP.*

#### *Who*

All authors agreed that the practitioner had the principal role in the phenomenon. Nevertheless, many insisted on the imbrication of the various stakeholders leading to inertia, and on the patient and health system responsibilities. O’Connor *et al*. proposed a conceptual model that combined physician, patient, and office and system factors [28]. The same type of classification emerged from the qualitative study of Howe *et al*., with some overlapping of the categories [29].

Although Phillips, in his initial article, considered that: “*Patient nonadherence cannot explain the failure of providers to initiate or advance therapy appropriately*”, he also admitted that: “*Clinical inertia may also reflect patients’ lack of enthusiasm for management of asymptomatic problem*” [10]. The actual complexity of the relation between the caregiver’s inertia and the patient’s adherence or preferences was often discussed: “*the inability to achieve adequate BP control likely arises through a complex interaction of patient and provider behaviors”*[30]. In their attempt to provide a conceptual model for clinical inertia, O’Connor *et al*. hypothesized that the various patient factors involved accounted for 30% of the whole phenomenon [28]. These factors would include denial of disease, low health literacy, number, cost and side effects of medications, and doctor-patient relationship issues. Lin *et al*. found that patient’s non-adherence was cited by the physician as the barrier to intensifying therapy in 19% of the visits, and “other patient factors” in 49% [31]. Reach proposed a common mechanism leading to physician’s inertia and patient nonadherence [25]. He defined “*clinical myopia*” as giving preference to the immediate and tangible benefits of nonadherence or inertia, instead of long-term benefits, and hypothesized that these behaviors, sharing the same psychological structure, enter into resonance. However, the large retrospective cohort study of Heisler *et al*. found that patient adherence had little effect on provider’s decision about intensifying therapy [32].

Office and system factors accounted for 20% of clinical inertia according to O’Connor *et al.*[28]. In the qualitative studies, time was an issue raised by many participants, and systematically related to competing demands [29,33] . Some authors agreed with O’Connor to regard this time issue as being a part of clinical inertia and include it in the “Physician factors”: “*Providers often have competing interests, including lack of time, more urgent requests made by the patient, and practice habits that can prohibit the escalation of care when such a modification is clinically indicated. This behavior (or lack thereof) is known as clinical inertia”*[34]*.* Others considered it as being out of the reach of the practitioner, and therefore not a part of clinical inertia: “…*health system issues such as lack of time in consultations.”*[35]; “*The impact of the medical environment should also be underscored (…) providers need to have adequate time and resources to be able to adhere to guidelines and to provide the necessary patient education and counseling*” [36].

#### *How and why*

Clinical uncertainty regarding BP measurements was considered in very different ways. Repeated measurement could be regarded as a need, as stated by general practitioners in a qualitative study: “*To monitor therapy more accurately, more automated machines for home monitoring and greater access to ambulatory BP monitoring were considered of need*” [29], or as a pure waste of time, according to Phillips and Twombly answering to criticism on their editorial: “*Our understanding of the basis for clinical inertia has been advanced by the demonstration of contributions from “clinical uncertainty” and “competing demands”, but it’s been almost 7 years since the concept was promulgated. We believe that rather than doing further studies on mechanisms, it’s time to focus on overcoming clinical inertia*” [37,38].

Acceptable control seemed to have two different acceptations. The first one was to consider that a BP close enough to the recommended target was satisfying, and the other that the actual target for a given patient would be dictated by the baseline BP [39-42]. Although most authors considered this behavior as inappropriate and unjustified, some had slightly different views. Banegas *et al*. pointed out that: “*In fact, the trial-based differences in achieved cardiovascular protection within this range of BP values seem to be small at best*” [43]. Discussing their empirically derived model of “clinical inaction” Safford et al. noted that: “*best level of control may appropriately differ from patient to patient as patients increase in complexity, especially in the geriatric population*” [44]. Others clearly stated that this behavior was not inertia. Crowley *et al*. conclude their work on hypertension telemanagement with: “*However, when physicians did not intensify treatment, it was because blood pressure was closer to an acceptable threshold, and repeat blood pressure elevations occurred less frequently. Failure to intensify treatment when home blood pressure is elevated may, at times, represent good clinical judgment, not clinical inertia*” [41], and Kennedy and Mac Lean stated: “*It is important to distinguish clinical inertia from modified therapeutic goals*” [45].

Competing demands have proven to contribute consistently to clinical inertia [11,12,46]. In terms of concepts, a controversy between authors summarizes the problem. Phillips and Twombly proposed in an editorial to overcome the problem by recommending that physicians *“run the numbers first and deal with blood pressure and glucose before asking about other problems”*[37]*.* This editorial led to a number of answers. Among them, Boyd and Leff stated that *“this is the wrong way to frame the issue because it does not adequately acknowledge a patient-centered perspective of chronic illness care, in which all of the patient’s conditions are considered in terms of the relative benefit of treating each condition in the presence of the other conditions, the cumulative effect of all the recommended treatments, and the individual’s treatment priorities”*[38]. Vijan *et al*. added that: “*If primary care physicians focused on the numbers first, they would end up imposing their own priorities onto patients, rather than letting patients help set the agenda. Consider a visit with a patient who has depression or chronic pain. Until a physician addresses such issues, there is little chance of managing chronic conditions well”*[38]*.*

Guidelines skepticism has been widely discussed in a number of conditions, including hypertension, since Cabana *et al*. founding article [47]. This skepticism includes distrust of the evidence underpinning the guidelines, discrepancies between the various guidelines, unrealistic treatment targets and inappropriateness for primary care. Each of these factors is controversial: *“clinical inertia may be a clinical safeguard through which physicians acknowledge the uncertainty in some current practice guidelines”*[22]; “*realistic expectations about the results of adherence to clinical practice guidelines are also called for when considering the subject of possible clinical inertia.*” [20]; “*clinical inertia or inaction may actually act as a safeguard for some patients when overzealous guidelines require treatment before definitive trials are available”*[19]; “*in most guidelines, the full versions make clear that evidence on targets is limited and their recommendations are unattainable in many patients”*[48]*.* Borzecki *et al*. separated guidelines skepticism from clinical inertia: “*The most important provider-related barriers to adherence to best practice include clinical inertia and lack of provider agreement with guidelines*” [36].

Overestimation of care is a well-known phenomenon. All authors agreed to consider its results as “pure inertia” that should be specifically and systematically addressed [49,50].

Perceived patient attitude, and notably perceived non-adherence or unwillingness to take more medications or to follow counseling, relates to both non-adherence and doctor-patient relationship. Although cited in many articles as a cause of inertia, it was very rarely explored, and even less commented. Campbell made this remark in an editorial about hypertension guidelines: “*Individual patients vary widely in their perception of acceptable risk and side effects.(…) Surprisingly, the patient's role in deciding his or her own blood pressure target receives scant attention in guidelines for hypertension*” [48].

#### *Appropriateness*

A number of authors insisted that the lack of treatment intensification for a patient who did not reach the target BP could actually reflect appropriate care. Various specific situations involving this issue were already highlighted in the previous sections. The gap between guidelines and actual care could be regarded as an appropriate translation of trials results in real-life: “*Sometimes the inertia may be appropriate. There might be a difference between effects in controlled trials and effectiveness in primary care patients. The GP has to take into account all circumstances for each patient, e.g. other risk factors, concurrent disease, medications, and function of different organs*” [51]; “*It is possible that the guidelines may be correct, but there is also the possibility that the care by the physicians is appropriate since BP 130/80 mmHg is hard to achieve, and recent reviews suggest there is insufficient evidence to support such a low BP target*” [43]. Hicks *et al*. conducted a prospective survey on the point of care in hypertensive diabetic patients. They found that: “*26% of patients are ‘near goal’, and action in this group is infrequent. This phenomenon has been referred to as ‘clinical inertia’ (…). The reasons given by providers for no action may reflect an individualized approach to patient care, rather than an unquestioned adherence to guidelines. (…)We did not find evidence for a pattern of a poor quality of care. On the contrary, providers seemed willing to consider the needs of their patients and the specific clinical circumstances*” [46].

With a nominal group approach, Safford *et al*. were able to provide some qualitative evidence on the possible appropriateness of inaction. Experts voted and agreed on a number of situations where inaction would be appropriate and others on when it would not. After giving useful clues for further research on the topic, the authors emphasized the need to make the appropriate decision “to not intensify treatment” as clear as possible: “*Distinguishing potential clinical inertia from appropriate inaction is an important initial step for interventionists seeking to identify strategies to improve care and for policy makers seeking to measure quality of health care*” [44].

## Discussion

### Main findings

This review retrieved major discrepancies between the authors regarding definition and conceptualization of therapeutic inertia. Opinions differed widely on every issue, from semantics to the inner quality of inertia regarding therapeutic decisions. Whereas some claimed that the practitioner’s decision should rely on numbers and numbers only, others regarded inertia as a choice that could sometimes be reasonable and adequate. On a more factual level, some suggestions for interventions to reduce clinical inertia went against the principles of evidence-based medicine and patient-centered practice, and some studies highlighted behaviors that matched the definition of therapeutic inertia but were nevertheless appropriate.

The reliability of BP measurement is crucial. Nested in therapeutic inertia as one of its factors, “clinical uncertainty”, defined as the feeling of the physician that the numbers might not be reliable and therefore the patient might not be hypertensive, appears to be a concept in itself. The diagnosis of hypertension means a lifelong treatment. The decision to initiate or intensify a treatment requires “certainty”, and emergency is exceptional. Whether a reasonable delay to secure a diagnosis with ambulatory measurement is acceptable is not addressed by the initial definition. In some trials and surveys, such a delay was regarded as pure inertia. Recent guidelines have advocated the systematic use of home or ambulatory blood pressure monitoring before any treatment initiation or modification [6]. On the other hand, a delay in confirmation should remain reasonable, and measurements should not be repeated indefinitely.

Above all, evidence-based practice in primary care should always remain patient-centered. Treating hypertension in a patient is a matter of numbers. Treating a hypertensive patient requires a thorough analysis of the patient’s global health and comorbidities, including psychological and social issues, and shared decision making on the patient’s expectations as well as the biomedical data [4,52,53]. Still, there is a risk of abusive reference to the informal frames of evidence-based practice and shared decision making to mask unjustified delays.

Our findings suggest that the definition of therapeutic inertia should take into account the inherent complexity of primary care situations. Health care system realities on one side, patients’ values and attitudes on the other, both interact with the GPs’ behaviours to generate a complex system, not accounted for by the initial definition. What is more, this definition did not take into account the consequences, deleterious, neutral or useful of inaction.

**Figure 2:**
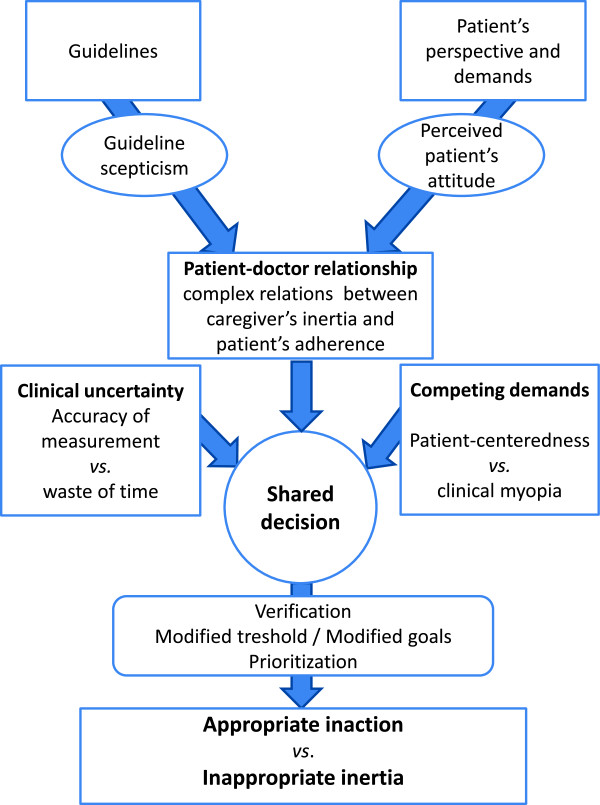
Model of shared decision-making leading to either appropriate inaction or inappropriate inertia.

A definition that merges an unacceptable loss caused by lack of knowledge, conviction, or time with a legitimate demand for reliable data and an appropriate decision is definitely not an operational definition. There is no sense in trying to reduce a complex phenomenon without knowing the precise conditions of its occurrence and to what extent it can be deleterious or useful. This issue has become increasingly acknowledged in the most recent articles, with a number of authors trying to differentiate “pure” inertia from “appropriate” inaction.Finally, our main conclusion is that it all comes down to appropriateness, with regards to both the various mechanisms of inertia and the patient-centered model of care (Figure 2).

### Further research

Semantics should now reflect these findings as clearly as possible. The words appropriate and inappropriate refer to neutrality and objectivity, without any judgment quality or manichaeism attached to them, and reflect a genuinely factual approach. We therefore suggest that two different definitions, one for “appropriate inaction” and the other for “inappropriate inertia”, should now be developed. Of course, we realize that a number of items in this review could be part of either of these two definitions. For example, clinical uncertainty can lead to appropriate inaction when blood pressure has been measured only in the office, and the practitioner claims for a home or ambulatory measurement, or to inappropriate inertia when this has been already done twice and yet a third time is scheduled. Further research is needed to clarify and precisely define to what extent each of these items should be accounted for, and achieve a consensus that should rely as much as possible on an inductive basis and empirical data.

The intimate causes and reasons leading to such behaviors should also be explored thoroughly. There is a major lack of qualitative data in this field. There is no way behaviors can be changed without prior exploration of their ins and outs.

On these new bases, interventions could be designed and assessed to either encourage appropriate inaction or reduce inappropriate inertia.

### Strengths and limitations

We followed the PRISMA guidelines as much as possible, provided that a number of items relate to quantitative systematic reviews and meta-analysis, and therefore could not be considered for this qualitative review [14].

As already stated, “inertia” is not a MeSH term, which made the search a bit more difficult and “risky”, and it is possible that we missed articles discussing the concept using other terms. We tried to avoid this by elaborating a search algorithm as sensitive as possible to all aspects of the concept, and by paying special attention to the publications cited in references in the selected articles. Although our concern was general practice, we did not include any MeSH terms related to primary care. Narrowing the search with such terms would have resulted in a loss of a few articles of interest, which discussed theoretical aspects of guideline adherence or inertia regardless of the context of care.

Although relying on a systematic search of the literature, this research was not a meta-synthesis of qualitative research. We conducted a qualitative analysis of original articles that could be qualitative research, quantitative research or opinion papers. There is no standard method for this kind of research, and the choice of a constant comparison qualitative method can be questionable. Because we were only looking for elements of definitions and concepts, quality assessment of the studies described in the selected articles was not justified. Therefore, the selection of the relevant articles very much depended on the researchers’ opinions. We tried to minimize this bias with a systematic blinded selection process.

We did not systematically search for “grey” literature. Considering the lack of qualitative research in this review and the wide use of qualitative methods in theses and dissertations, we might have missed some interesting works. However, it is unlikely that such works would have dramatically modified the results.

When dealing with definitions and concepts, exploring the causes (“How and why”) might seem questionable, and even out of focus. But when it comes to intimate mechanisms of human behaviours, causes and consequences exist first, and then, possibly, the concept arises. A number of authors in this review did think about the definition and the concept starting from observed or assumed causes, and so their contribution to the conceptualization was in terms of (possible) causes, which justified the “How and why” section of the results.

Finally, the extraction of data and their coding is always, to some extent, affected by the personal understanding of the researcher. However, the two teams coded separately and a fifth researcher adjudicated any discrepancies, in order to minimize this bias.

## Conclusion

This systematic review of the literature revealed important discrepancies, and sometimes antagonisms, regarding the possible causes, inner mechanisms and outcomes of therapeutic inertia in hypertension. The initial definition proposed by Phillips, and referred to by most authors, does not take into account the inner complexity of doctor-patient relationship and shared decision making in primary care.

Our data analysis led us to conclude that the concept of therapeutic inertia should be split into two separate concepts, namely appropriate inaction and inappropriate therapeutic inertia. The development of consensual and operational definitions and the exploration of intimate mechanisms that underlie these behaviors are now needed.

## Competing interests

The authors declare that they have no competing interests.

## Authors’ contributions

All authors participated in the conception and initial design of the project. JPL, JSC and TP searched the databases and other sources. JPL, JSC, IAA, AM and TP assessed the articles, extracted the data, and performed the initial coding. ER, KH and EV reviewed the initial coding. All authors participated in definition and organization of the categories. JPL, KH and EV drafted the manuscript. All authors revised the draft and approved the final manuscript.

## Pre-publication history

The pre-publication history for this paper can be accessed here:

http://www.biomedcentral.com/1471-2296/15/130/prepub

## Supplementary Material

Additional file 1 Results. Click here for file
