# Supplementary material for: The concept and definition of therapeutic inertia in hypertension in primary care: a qualitative systematic review
Source: BMC Fam Pract. 2014 Jul 2;15:130. doi: 10.1186/1471-2296-15-130 (PMC4094689; doi:10.1186/1471-2296-15-130)
Supplement: Additional file 1 — Results. [file 1471-2296-15-130-S1.pdf]

Citation	Author	Title	Country	Publication type	Results
N Engl J Med 1998,**339:**1957–63.	Berlowitz	Inadequate management of blood pressure in a hypertensive population.	USA	Cross-sectional study: Examination of a database	*"Despite two years of care, with many opportunities to increase antihypertensive medications, blood pressure continued to be poorly controlled in many patients. Thus, although physicians may have been closely monitoring patients' blood pressure, they repeatedly delayed making changes in the regimen."* *"Inadequate control of blood pressure can no longer be ascribed solely to the lack of access to medical care and noncompliance with therapy; physicians themselves must accept some responsibility for the problem."*
Am J Manag Care 1998,**4**(Suppl 12):741–744.	Cabana	Barriers to guideline adherence. Based on a presentation by Michael Cabana, MD	USA	Expert opinion: Report of a presentation and roundtable of experts	Discusses possible reasons for poor implementation of guidelines, on a deductive basis: lack of awareness, lack of agreement, lack of self-efficacy, lack of outcome expectancy, lack of cueing mechanism.
JAMA 1999,**282:**1458–1465.	Cabana	Why don't physicians follow clinical practice guidelines? A framework for improvement.	USA	Review of the literature: barriers to physician adherence to clinical practice guidelines	Proposes a model of the barriers to adherence in relation to behavior change, regarding knowledge, attitudes, and behavior. *"Inertia of previous practice"* is identified as an attitude. *"A theoretical approach can help explain these barriers and possibly help target interventions to specific barriers."*
Fam Pract 2001,**18:**359–363.	Cranney	Why do GPs not implement evidence-based guidelines? A descriptive study.	UK	Qualitative study: Content analysis of focus goups of GPs	The concept of inertia as such is not discussed. Nevertheless, all possible causes for inertia are found in the verbatim, including: *"I'm sure that at 6 o'clock on Friday, I'm not that fussed whether it's 160 or 164-I just want to go home!"*
Ann Intern Med 2001,135:825–834.	Phillips	Clinical inertia.	USA	Expert opinion: Initial definition and description of a new concept, on a deductive basis.	*"Strong evidence now indicates that therapy for hypertension, dyslipidemia, and diabetes can prevent or delay complications. The goals for management are well defined, effective therapies are widely available, and practice guidelines for each of these diseases have been disseminated extensively. Despite such advances, health care providers often do not initiate or intensify therapy appropriately during visits of patients with these problems. We define such behavior as clinical inertia—recognition of the problem, but failure to act."* *"Experienced clinicians will recognize that exceptions always occur and rigid insistence on the uniform application of guidelines for patient management could result in overtreatment or inappropriate actions."*
Arch Intern Med 2003,**163:**2677–2678.	O'Connor	Overcome clinical inertia to control systolic blood pressure.	USA	Editorial	*"The most common mistake in chronic disease care is not prescribing the wrong drug or forgetting to check a creatinine or potassium level when indicated, it is failure to initiate or titrate medications until important evidence-based clinical goals are reached. One of the major obstacles to better BP control is clinical inertia. Clinical inertia may be simply defined as an office visit at which no therapeutic move was made to lower the BP of a patient with uncontrolled hypertension."* *"We fail to recommend intensified treatment for a variety of reasons, only some of which are legitimate."* *"We physicians are tempted to lay the blame for clinical inertia at the feet of our patients. Yet, there is accumulating evidence to suggest that approximately 75% of the time, physician inertia is the reason for the problem, and approximately 25% of the time, failure to intensify treatment is due to patient refusal or resistance."*
Am J Health-Syst Pharm 2004,**61:**401–404.	Kennedy	Clinical inertia: Errors of omission in drug therapy.	USA	Commentary: How to increase pharmacist awareness regarding clinical inertia.	*"We believe the manifestation of clinical inertia is a pattern of repeated errors of omission. An error of omission is 'a failure to carry out the necessary steps in the performance of a task'."*
Am Heart J 2005,**149:**785–794.	Borzecki	Barriers to hypertension control.	USA	Narrative review: Factors associated with impaired control of BP	*"The term clinical inertia describes the phenomenon whereby clinicians do not initiate or intensify therapy appropriately for patients with chronic medical conditions such as hypertension, diabetes, and dyslipidemia."* Separates CI from disagreement with guidelines: *"The most important provider-related barriers to adherence to best practice include clinical inertia and lack of provider agreement with guidelines"*
Am Heart J 2005,**149:**795–803.	Bosworth	Improving blood pressure control by tailored feedback to patients and clinicians.	USA	Narrative review	*"Research has attributed these findings to a tendency for clinicians to use a higher threshold of clinic-based blood pressure to initiate regimen changes ("clinical inertia")."* *" Other physician-related factors are not included in clinical inertia."*
In *Advances in Patient Safety: From Research to Implementation (Volume 2: Concepts and Methodology).* Edited by Henriksen K. Rockville: Agency for Healthcare Research and Quality; 2005:293-[illegible]	O'Connor	Clinical inertia and outpatient medical errors.	USA	Position paper: Propositions for a conceptual model, an operative definition, and future research	*"Clinical inertia is defined as lack of treatment intensification in a patient not at evidence-based goals for care.(...)In instances of clinical inertia, both of the following must occur: (a) The patient fails to achieve major evidence-based clinical goals, and (b) the patient fails to receive appropriate intensification of pharmacotherapy in a defined period of time."* *"To operationally define clinical inertia, several decisions must be made. First, the clinical goals of care must be selected. Second, the therapy of the disease must be defined in such a way that it can be measured. Finally, one must define a time window from the date of a visit, test, or other clinical event within which intensification of therapy is designated as timely.(...) Flexibility in how clinical inertia is defined could be seen by some as a limitation. However, from the point of view of care improvement, this sort of flexibility may often be an advantage because it allows local tailoring of initiative and interventions."*
J Clin Hypertens 2006,**8:**481–486.	Carter	Relationship between physician knowledge of hypertension and blood pressure [illegible]	USA	Cross-sectional study	*"This study demonstrates that there is no evidence that high knowledge of hypertension guidelines will improve BP control rates and that higher knowledge may actually be associated with lower BP control"*

Curr Med Res Opin 2006,**22:**1545–1553.	Düsing	Overcoming barriers to effective blood pressure control in patients with hypertension.	Germany	Literature review: Non-systematic Medline review	Does not use the word "inertia", but states that : *"Modifiable factors under the control of the physician include: (1) insufficient identification of hypertensive patients (e.g. by not strictly applying 140/90 mmHg as the age-independent cut-off blood pressure between normotension and hypertension); (2) failure to select the therapeutic options most appropriate for each patient; (3) uncertainty regarding when and how to implement lifestyle changes and when to initiate drug treatment; (4) providing insufficient information and failing to motivate patients to accept the need for lifestyle changes or drug treatment; (5) insufficient emphasis on the importance of lowering systolic BP levels to < 140 mmHg; (6) reluctance to modify therapy appropriately when BP goals are not achieved and (7) failure to follow up patients regularly with sufficient rigour to ensure that they are adhering to lifestyle advice (e.g. in achieving body weight control and reducing excessive alcohol or NaCl intake) and to the prescribed drug regimen."*
Diabetes Care 2006,**12:**2580-2585.	Hicks	Action or inaction? Decision making in patients with diabetes and elevated blood pressure in primary care.		Prospective survey study	*"In our study, 26% of patients are 'near goal', and action in this group is infrequent. This phenomenon has been referred to as 'clinical inertia'."*<br>*"The reasons given by providers for no action may reflect an individualized approach to patient care, rather than an unquestioned adherence to guidelines (…) it appears that balancing clinical decisions accounts for most instances of inaction, not knowledge deficits."*<br>*"From this study, it is clear that whereas blood pressures above national goals are common, the majority of patients are at or close to target. This finding suggests that primary care clinicians are actively and aggressively trying to control a major cardiovascular risk factor in their patients with diabetes. We did not find evidence for a pattern of a poor quality of care. On the contrary, providers seemed willing to consider the needs of their patients and the specific clinical circumstances, including how elevated the blood pressure is, and issues such as polypharmacy, medication side effects, and costs when determining the best course of action."*
AMIA Annu Symp Proc 2006:494–498.	Lin	Identifying Barriers to Hypertension Guideline Adherence Using Clinician Feedback at the Point of Care.	USA	Randomized clinical trial: Based on the Cabana model	*"Clinicians commonly reported they did not follow recommendations because: recorded blood pressure was not representative of the patient's typical blood pressure; hypertension was not a clinical priority for the visit; or patients were nonadherent to medications. These factors may describe appropriate clinician guideline non-adherence in certain patient scenarios."*
Hypertension 2006,**3:**345-351.	Okonufa	Therapeutic inertia is an impediment to achieving the Healthy People 2010 blood pressure control goals.	USA	Retrospective cohort study	*"Physician factors, such as therapeutic inertia (TI), that is, failure of providers to begin new medications or increase dosages of existing medications when an abnormal clinical parameter is recorded, are becoming more evident. TI represents a significant barrier to better hypertension control."*<br>*"Although data suggest that TI contributes to the high prevalence of uncontrolled hypertension, the quantitative impact is not clear."*<br>*"This limitation in the database prompted us to use the term TI, given our focus on medication change rates, rather than clinical inertia, which implies a more comprehensive analysis of the management plan."*
J Clin Hypertens 2006,**8:**667–670.	Pickering	Therapeutic inertia and the Medicare crisis.	USA	Expert opinion	*"blood pressure >140/90 mm Hg resulted in no change of medications being recommended by the treating physician, a phenomenon referred to as therapeutic inertia (TI)."*<br>*"TI is one critical ingredient that distinguishes a clinical trial from routine practice"*
Curr Hypertens Rep 2006,**8:**324–329.	Ruzicka	Moving beyond guidelines: are report cards the answer to high rates of uncontrolled hypertension?	Canada	Narrative review	*"The term "therapeutic inertia" was coined to define a failure to intensify BP-lowering therapy despite the opportunity to do so and despite the overwhelming evidence for benefits to the patient from doing so."*<br>*"Physicians may also rationalize lack of action caused by disagreement with guidelines. (...) lack of data from large randomized clinical trials on those with mild isolated systolic hypertension (systolic BP 140–159 mm Hg) may contribute to the therapeutic inertia toward older patients."*
Arch Intern Med 2006,**166:**507–513.	Ziemer	An intervention to overcome clinical inertia and improve diabetes mellitus control in a primary care setting: Improving Primary Care of African Americans with Diabetes (IPCAAD)	USA	Randomized clinical trial: reminders and/or feed-back vs control in diabetes	*"the failure of health care providers to initiate or intensify therapy appropriately—clinical inertia"*
J Clin Hypertens 2007,**9:**113–119.	Ardery	Explicit and implicit evaluation of physician adherence to hypertension guidelines.	USA	Retrospective cohort study	*"Clinical inertia, that is, the failure to promptly initiate/ adjust therapy and follow-up with patients who had abnormal clinical BPs."*<br>*"Infrequent documentation of lifestyle recommendations could, however, reflect another type of clinical inertia—namely, missed opportunities to promote patient self-management."*<br>*"The most frequently identified factors were the presence of comorbidities and patient noncompliance."*
J Clin Hypertens 2007,**9:**636–645.	Basile	The role of therapeutic inertia and the use of fixed-dose combination therapy in the management of hypertension.	USA	Expert opinion	*"And I think that they [patients] bargain with their doctor and talk their doctor into therapeutic inertia, seeing a pressure that's elevated and doing nothing about it."*<br>*"But I think it is a huge term [clinical inertia] that includes many different things."*
Fam Pract 2007,**24:**547–554.	Bebb	Practice and patient characteristics related to blood pressure in patients with type 2 diabetes in primary care: a cross-sectional study.	UK	Cross-sectional study	*"Older people with hypertension are managed differently than younger people with hypertension, in terms of frequency of follow-up and the classes of drugs prescribed.(...) GPs and practice nurses are more reluctant to aggressively treat hypertension in elderly patients. "*<br>*"The only practice factor we found to be significantly associated with blood pressure control was negotiating targets with patients. "*

Fam Pract 2007,**24:**259–262.	Dean	Pilot study of potential barriers to blood pressure control in patients with inadequately controlled hypertension.	UK	Cross-sectional study	*"'clinical inertia'—reluctance to change treatment despite failure to achieve target BP and lack of regular review."*
Br J Gen Pract 2007,**57:**948–952.	Heneghan	Hypertension guideline recommendations in general practice: awareness, agreement, adoption, and adherence.	UK	Descriptive survey study	*"Non-implementation of guidelines may be due to several factors: lack of awareness, lack of agreement, lack of belief that one can actually perform a behaviour, lack of expectation that a given behaviour will lead to a particular consequence, the inertia of previous practice, and external barriers."*
Sang Thrombose Vaisseaux 2007,**9:**181–182.	Mourad	Blood pressure control and therapeutic inertia in the HTA (arterial hypertension). French, European and North American	France	Commentary: On: Wang et al. Arch Intern Med 2007;167:141-7.	[Translated from french]*"Therapeutic inertia (defined as lack of treatment intensification or modification during the consultation in a patient not at goal)..."*
Ann Fam Med 2007,**5:**196–201.	Parchman	Competing demands or clinical inertia: the case of elevated glycosylated hemoglobin.	USA	Cross-sectional study: Analyzes the link between prescriptions of glucose-lowering agents, HbA1C, and symptoms or complaints expressed by the patient	*"The phenomenon of clinical inertia has been diffi cult to study because of the paucity of data on the content of the patient-physician encounter.(...) An alternative explanation for failure to intensify therapy despite poor glucose control is the presence of competing demands."*<br>*"The concept of clinical inertia is limited and does not fully characterize the complexity of primary care encounters. Competing demands is a principle for constructing models of primary care encounters that are more congruent with reality and should be considered in the design of interventions to improve chronic disease outcomes in primary care settings."*
Jt Comm J Qual Patient Saf 2007,**33:**277–285.	Roumie	Clinical inertia: a common barrier to changing provider prescribing behavior.	USA	Cross-sectional study	*"Providers often have competing interests, including lack of time, more urgent requests made by the patient, and practice habits that can prohibit the escalation of care when such a modification is clinically indicated. This behavior (or lack thereof) is known as "clinical inertia."(...) the failure to initiate or titrate medications as needed to reach important goals."*<br>*"The negative responses were categorized as: inertia of practice (66%), lack of agreement with specific guidelines (5%), patient-based factors (17%), environmental factors (10%), and lack of knowledge (2%). "*<br>*"Many providers have higher blood pressure thresholds for diagnosis and treatment of hypertension than the 140/90 recommended."*<br>*"A comment that was categorized into systems barriers is: 'Patient has been primarily followed by an outside cardiologist'."*
J Gen Intern Med 2007,**22:**1648–1655.	Safford	Reasons for not intensifying medications: differentiating "clinical inertia" from appropriate care.	USA	Nominal group consensus	*" Clinical inertia is a recently described phenomenon of physicians failing to intensify medication regimens at encounters with patients who have uncontrolled risk factors. Our findings suggest that many such apparent "failures" to intensify medication regimens reflect potentially appropriate decisions in many cases. These findings suggest that part of the explanation for previously reported low intensification rates is appropriate inaction. (...) Distinguishing potential clinical inertia from appropriate inaction is an important initial step for interventionists seeking to identify strategies to improve care and for policy makers seeking to measure quality of health care."*
Nat Clin Pract Endocrinol Metab 2007,**3:**452–453.	Turchin	Is clinical inertia a common barrier to patient care in type 2 diabetes mellitus?	USA	Commentary	*"Lack of treatment intensification in the face of abnormal clinical findings—sometimes referred to as 'clinical inertia'."*<br>*"Physicians who participated in the study felt that blood pressure was "at or near goal" or "was improving", believed the elevation in blood pressure to be "transient", or felt that the focus of the visit was for a clinical problem separate from blood pressure control. (...) reasons for inaction differed depending on the blood pressure level."*
Hipertension 2007,**24:**91–92.	Vinyoles	Not only clinical inertia...	Spain	Editorial	[Translated from spanish] *"Clinical inertia, that is our reluctance to make changes in the treatment."*<br>*"Three inertias are barriers to change: physician's inertia, patient's inertia, and health authorities inertia."*
Health Aff (Millwood) 2007,**26:**899–900.	Wexler	Clinical inertia and organizational change.	USA	Letter	*"'clinical inertia', which they describe as 'recognition of the problem but failure to act'."*
Rev Clin Esp 2008,**208:**393–399.	Alonso-Moreno	[Primary care physicians behaviour on hypertensive patients with poor blood pressure control. The PRESCAP 2006 study].	Spain	Cross-sectional study	[Translated from spanish] *"The passive behaviour of a practitioner facing a situation that would require treatment modification according to evidence and guidelines is called clinical inertia."*
J Hum Hypertens 2008,**22:**63–70.	Bakris	Achieving blood pressure goals globally: five core actions for health-care professionals. A worldwide call to action.	USA	Expert opinion	*"Clinician inertia, whereby physicians and other clinicians treating hypertension are unwilling to increase the intensity of drug treatment even though they see patients regularly and are aware that blood pressure goals have not been achieved."*
Adv Ther 2008,**25:**300–320.	Ferri	Role of combination therapy in the treatment of hypertension: focus on valsartan plus amlodipine.	Italy	Literature review	*"Therapeutic inertia deriving from poorly prescribed Lifestyle changes, excess monotherapy use, and scarce on-treatment modifications."*
Circulation 2008,**117:**2884–2292.	Heisler	When more is not better: treatment intensification among hypertensive patients with poor medication adherence.	USA	Retrospective cohort study: Relationship between patient adherence and provider treatment	*"When faced with elevated BP, providers often do not appropriately increase medication dose or number of medications(...). Such failures to intensify medications, often labeled "clinical inertia," are associated with poor BP control."*<br>*"Patients' prior medication adherence had little impact on providers' decisions about intensifying medications, even at very high levels of poor adherence."*

Dis Manag<br>2008,**11:**71–77.	Holland	Identifying barriers to hypertension care: implications for quality improvement initiatives.	USA	Cross-sectional study	*"The failure of health care providers to intensify medication regimens despite patients not achieving treatment goals is often referred to as 'clinical inertia' or 'therapeutic inertia'."*
Ann Intern Med<br>2008,**148:**717–727.	Kerr	The role of clinical uncertainty in treatment decisions for diabetic patients with uncontrolled blood pressure.	USA	Prospective cohort study	*"'clinical inertia'—the failure by providers to initiate or intensify therapy (medication intensification) in the face of apparent need to do so—is a main contributor to poor control of hypertension."*<br>*"Clinical uncertainty about the true blood pressure value was a prominent reason that providers did not intensify therapy."*
Hipertension<br>2008,**25:**187–193.	Marquez Contreras	Clinical and professional inertia and drug non-compliance. How do they influence control of hypertension? The CUMAMPA study.	Spain	Longitudinal study	[Translated from spanish]*"Clinical therapeutic inertia is defined as failure of the practitioner to diagnose, or to initiate or increase therapy when indicated."*
Scand J Prim Health Care<br>2008,**26:**154–159.	Midlöv	Barriers to adherence to hypertension guidelines among GPs in southern Sweden: a survey.	Sweden	Descriptive survey study	*"Clinical inertia is defined as recognition of the problem but failure to act, which is possibly related to overestimation of care provided, use of ''soft'' reasons to avoid intensification of therapy, or lack of training."*<br>*"Sometimes the inertia may be appropriate. There might be a difference between effects in controlled trials and effectiveness in primary care patients. The GP has to take into account all circumstances for each patient, e.g. other risk factors, concurrent disease, medications, and function of different organs."*
J Clin Hypertens<br>2008,**10:**822–829.	Nwachuku	Management of high blood pressure in clinical practice: Perceptible qualitative differences in approaches utilized by clinicians.	USA	Qualitative study	*"Physicians are not as aggressive as they should be in their willingness to intensify medical therapy for the elderly—so-called clinical inertia."*
J Clin Hypertens<br>2008,**10:**644–646.	Ogedegbe	Barriers to optimal hypertension control.	USA	Narrative review	*"Clinical inertia, which is defined as the failure of health care providers to initiate or intensify drug therapy appropriately in a patient with uncontrolled BP."*
Ann Intern Med<br>2008,**148:**783–785.	Phillips	It's time to overcome clinical inertia.	USA	Editorial	*"Blood pressure levels remain above goal because providers do not initiate or intensify therapy when clinically indicated. We have characterized this problem as "clinical inertia"."*<br>*"Competing demands contribute less consistently to clinical inertia than clinical uncertainty. (...) Clinical inertia is not linked to patient sex or race."*
Diabetes Metab<br>2008,**34:**382–385.	Reach	Patient non-adherence and healthcare-provider inertia are clinical myopia.	France	Theoretical model based on concepts of analytical phylosophy of mind	*"Lack of adhernece of the healthcare provider to current guidelines, a relatively new concept referred to as 'clinical inertia'"*
J Hypertens Suppl<br>2008,2**6:**S1–S14.	Redon	Practical solutions to the challenges of uncontrolled hypertension: a white paper.	Spain, switzerland, Italy, Germany, Netherland	Expert opinion	*"The profession may be paralysed by the bewildering amount of information and therapeutic inertia is setting in."*<br>*"A therapeutic inertia score was dertermined by calculating the difference between expected and observed medication rates."*<br>*"The working group identified six challenges that stand in the way of achieving goal blood pressure for all patients; an urgent need of simplicity; therapeutic inertia; lack of empowerment and responsibility; and unsupportive healthcare structures and policy."*
J Gen Intern Med<br>2008,**23:**180–183.	Rose	The accuracy of clinician perceptions of "usual" blood pressure control.	USA	Cohort study	*"The term "clinical inertia" is used to describe the failure to manage a chronic condition aggressively enough to bring it under control."*<br>*"Clinicians may not be aware of or agree with consensus guidelines regarding BP targets. (...) Clinicians may have access to data not available from the vital signs module of the EMR (electronic medical record)."*
Ann Intern Med<br>2008,**149:**838–841.	Letters, comments and responses (various authors)	Will running the numbers first violate the principles of patient-centered care?	USA	Letter:<br>Comments on Phillips et al.: It's time to overcome clinical inertia	*"Defining clinical inertia as a failure is pejorative. On the one hand, it may be that physicians are not treating an important problem as effectively as possible. Alternatively, physicians may be providing patient-centered care, accounting for patients' individual situations and multimorbidity."*<br>*"Phillips and Twombly recommend that every time blood pressure is elevated, clinicians should intensify therapy. This approach promotes potentially harmful polypharmacy, given that most patients with diabetes require at least 2 to 3 blood pressure medications, and it also increases the risk for nonadherence due to side effects and cost."*<br>*"Rather than suffering from clinical inertia, clinicians may be simply following the guidelines. (...) Our understanding of the basis for clinical inertia has been advanced by the demonstration of contributions from "clinical uncertainty" and "competing demands"."*
Ann Intern Med<br>2008,**148:**578–586.	Turner	Effect of unrelated comorbid conditions on hypertension management.	USA	Retrospective cross-sectional study	*"clinical complexity, as reflected by unrelated comorbid conditions, should be considered when evaluating quality of care."*

J Managed Care Pharm 2009,**15:**690–695.	Allen	Nonadherent, clinical inertia, or therapeutic inertia?	USA	Editorial	*"'Clinical inertia' and 'therapeutic inertia' have been used recently by authors, primarily to attribute to physicians the apparent failure of patients to attain therapeutic blood pressure goals."* *"Clinical inertia is an important theoretical construct that encompasses the underuse of therapy that is efficacious and effective in preventing serious endpoint clinical outcomes such as death, nonfatal myocardial infarction (MI), and stroke.* *"Clinical inertia occurs when health care providers recognize the problem (failure to attain therapeutic targets in patients with hypertension, dyslipidemia, or diabetes) but fail to act (to initiate or intensify therapy). Clinical inertia must also be evaluated in the context that evidence-based practice is a moving target."* *"Realistic expectations about the results of adherence to clinical practice guidelines are also called for when considering the subject of possible clinical inertia. (...) we suggest that when this term is used that authors be specific and not attribute failure of patients to meet therapeutic goals solely to clinicians failure to intensify treatment in a timely manner. "*
J Clin Hypertens 2009,**11**(Suppl 1):5–12.	Basile	Clinical inertia and blood pressure goal attainment.	USA	Narrative review	*"Most clinicians generally do not take an aggressive enough approach in their treatment of hypertension. In essence, they are guilty of therapeutic inertia."* *"Therapeutic inertia was defined as systolic BP >140 mm Hg and ⁄ or diastolic BP >90 mm Hg, with no change in antihypertensive therapy."*
Nephrol Ther 2009,**5**(Suppl 4):240–245.	Bobrie	[From clinical observation to assessment of practices: guidelines for hypertension management].	France	Narrative review	[Translated from french] *"'Clinical inertia', the failure to initiate or change therapy when indicated."*
Am Heart J 2009,**157:**450–456.	Bosworth	Patient education and provider decision support to control blood pressure in primary care: a cluster randomized trial.	USA	Randomized clinical trial	*"Physicians may be reticent to increase the intensity of pharmacologic management of patients when BP approaches target values (ie, "clinical inertia"). Although the root causes of this are not known, log summaries from clinicians using the system provide insight into the barriers perceived by the clinicians, including the clinic BP not representative of the patient's typical BP, hypertension not the clinical priority for that visit, and patient nonadherence to medication."*
J Am Soc Hypertens 2009,**3:**267–276.	Faria	A narrative review of clinical inertia: focus on hypertension.	USA	Narrative review	*"Clinical inertia (CI) is defined as the inappropriate management of at least one medical condition for a given patient because of minimal or lack of appropriate therapeutic intervention. (...) failure to act despite recognition of a problem with a known solution."* *"Theoretically, the existence of CI is inversely related to therapeutic modifications."*
J Hum Hypertens 2009,**23:**151–159.	Ferrari	Reasons for therapeutic inertia when managing hypertension in clinical practice in non-Western countries.	Australia, switzerland	Prospective survey study	*"Failure of physicians to follow guidelines is apparently dependent on the belief that baseline BP dictates the target, that a clear improvement in BP might be sufficient and that the full drug effect may take up to 4 months or more to be attained."* *"Issues on the compliance of the patients accounted for approximately 10% of the reasons for not intensifying antihypertensive treatment throughout the study, whereas modification of other risk factors (smoking, lipid disorders, overweight, etc.) was used as an argument in 5% of cases."*
Arch Cardiovasc Dis 2009,**102:**465–467.	Lantelme	Blood pressure control: time for action.	France	Editorial	*"It must be remembered that guidelines have to be interpreted for each individual patient. In this respect, it is rather reassuring that the general health status is taken into account and antihypertensive treatment adapted accordingly. Longterm preventive treatment is indeed only conceivable if there is no other rapidly lethal disease."*
Aten Primaria 2009,**41:**315–323.	Márquez Contreras	[Control of therapeutic inertia in the treatment of arterial hypertension by using different strategies].	Spain	Randomized clinical trial	*"Therapeutic inertia (TI) is defined as the failure of the doctor or nurse in the initiation or maintenance treatment of a disease or risk factor when they are actually given, and despite knowing that following the protocols and practice guidelines is necessary do so. TI is a conservative attitude of professionals to the therapeutic decisions in managing different clinical situations that arise daily patients. It is a difficult concept to explain and justify. The reasons adduced are diverse, and among them include a lack of training, lack of confidence in the consensus, the lack of time in consultations and the complicated structure and health organization."*
J Clin Hypertens 2009,**11:**1–4.	Moser	Physician or clinical inertia: what is it? Is it really a problem? And what can be done about it?	USA	Editorial	*"Physician inertia is defined as the failure to initiate therapy or to intensify or change therapy in patients with BP values >140 ⁄ 90 mm Hg, or >130 ⁄ 80 mm Hg in hypertensive patients with diabetes, renal, or coronary heart disease. The term clinical or physician inertia has been used to describe situations in which patients return for visits having taken their medication but have not had therapy changed despite BP levels that are higher than levels established by guidelines. It has also been applied with regard to the large number of patients (usually older than 60 years) with systolic hypertension for whom physicians are reluctant to provide any specific treatment."* *"Some inertia is also the result of confusion as to which BP measure to use. Data suggest that some physicians are confused: should clinic or home BP readings be used for treatment decisions? Should ambulatory BP monitoring be performed to get the ''true'' BP?"*
Hypertens Res 2009,**32:**753–758.	Rodriguez-Roca	Blood pressure control and physicians' therapeutic behavior in a very elderly Spanish hypertensive population.	Spain	Cross-sectional study	*"Mistaken physician perceptions about BP control for their patients dramatically increased (OR: 108.1) the probability of not making changes in the antihypertensive treatment of participants with uncontrolled BP. (...) an incorrect perception of BP control may be one of the most important factors for therapeutic inertia."*
Hipertension Riesgo Vasc 2009,**26:**98–104.	Sanchis Domenech	"Kontrol objective" study: therapeutic inertia in arterial hypertension. Design and methodology.	Spain	Qualitative study	[Translated from spanish] *"This attitude is known as therapeutic inertia, defined as the failure of the practitioner to initiate or intensify therapy when indicated."*
Fam Pract 2009,**26:**428–436.	van Bruggen	Clinical inertia in general practice: Widespread and related to the outcome of diabetes care.	Netherland s	Randomized clinical trial	*"This failure to initiate or intensify therapy when indicated has been called clinical inertia."* *"According to the GPs, prescribing could be influenced by patients' reluctance to start or continue pharmacotherapy "*

Patient 2009,**2:**221–231.	Zikmund-Fisher	First things first: difficulty with current medications is associated with patient willingness to add new ones.	USA	Cross-sectional study	*"Clinical inertia, often defined as the failure by providers to initiate or intensify medication therapy when otherwise appropriate. However, patients could contribute to clinical inertia by signaling an unwillingness to consider medication intensification."*
Ther Adv Cardiovasc Dis 2010,**4:**119–127.	Basile	Overcoming clinical inertia to achieve blood pressure goals: the role of fixed-dose combination therapy.	USA	Literature review	*"Clinical or therapeutic inertia is defined as the providers' failure to begin new medications or increase dosages of existing medications when treatment goals remain unmet. Clinical inertia can occur even when patients have demonstrated compliance with their therapy . It has also been defined as a failure to act despite recognition of the problem. "* *"Undertreatment and clinical inertia can also be mistaken for treatment-resistant hypertension."*
Postgrad Med 2010,**122:**35–48.	Basile	Identifying and managing factors that interfere with or worsen blood pressure control.	USA	Expert opinion	*"A practitioner's failure to intensify treatment despite evidence of poor BP control is known as therapeutic inertia."*
Vasc Health Risk Manag 2010,**6:**321–325.	Düsing	Optimizing blood pressure control through the use of fixed combinations.	Germany	Literature review	*"Doctors are often hesitant to expand therapy in treated patients whose blood pressure is not lowered to goal (therapeutic inertia)"*
J Natl Med Assoc 2010,**102:**1231-1236.	Fiscella	A novel approach to quality improvement in a safety-net practice: concurrent peer review visits	USA	Randomized trial	*"Competing visit demands and clinician inertia (ie, failure to initiate change in treatment when clinically indicated) during 15-minute visits make it difficult for clinicians to focus on achieving these targets. Cognitive overload may also contribute to implicit clinician bias (ie, activation of unconscious stereotypes that affect clinical behavior), particularly in underresourced practices where working conditions may be especially stressful."*
Blood Press 2010,**19:**3–10.	Gil-Guillén	Clinical inertia in diagnosis and treatment of hypertension in primary care: quantification and associated factors.	Spain	Cross-sectional study	*"Inertia was defined if a patient showed high BP according to clinical guidelines and the physician failed to act upon it. Diagnostic inertia was defined as a failure to consider the diagnosis of HTN in a subject in the absence of diagnosis of HTN and elevated BP. On the other hand, therapeutic inertia was defined in an uncontrolled hypertensive if therapeutic action was not taken."*
Arq Bras Cardiol 2010;**95:**223–229.	Hoepfner	Therapeutic inertia and control of high blood pressure in primary health care	Brazil	Cross-sectional study	*"Therapeutic inertia, i.e., the failure of health professionals to initiate or intensify a therapy when this is indicated."* *"Just as the therapeutic inertia may be overestimated, the comorbidities may be underestimated"*
Aust Fam Physician 2010,**39:**511–516.	Howes	Barriers to diagnosing and managing hypertension - a qualitative study in Australian general practice.	Australia	Qualitative study	*"Clinical inertia is the recognition of a problem and the failure to act and it has been described as an issue in the management of patients with asymptomatic chronic illnesses such as hypertension, dyslipidaemia and diabetes. "* *"Reluctance to initiate treatment in someone else's patient' despite repeated high BPs recorded."* *"Failure to reach target was further justified by discussing BP as a continuum, with small improvements viewed as a therapeutic success: 'In my own mind I just sort of figure, well if their BP is below 140/80 it's not too bad, and if they're diabetic ideally you should be pushing it further down but I find I get a bit lazy once it reaches that sort of level and I'm less likely to pursue it'."* *"General practitioners described a process of 'mental adjustment' of BP readings. They were adjusted down to 'better represent' what was thought to be the patient's 'true' underlying BP."* *"General practitioners agreed that the negative impact on patient quality of life may outweigh the benefits of treatment."*
Rev Med Liege 2010,**65:**273–277.	Krzesinski	[Therapeutic inertia in hypertension: why and how to fight against this attitude?].	Belgium	Narrative review	[Translated from french] *"Therapeutic inertia is the lack of initiation or intensification of therapy when blood pressure goals are unmet."* *"As for therapeutic inertia, the responsability lies in the practitioner's behaviour, continuing on his initial decisions."* *"There is a strong link between therapeutic inertia and awareness of cardiovascular risk, poor blood pressure control, and goals*
Rev Med Liege 2010,**65:**256–260.	Suarez	Clinical inertia in geriatrics.	Belgium	Narrative review	[Translated from french] *"Numerous causes lead to therapeutic inertia, i.e. 'lack of initiation or intensification of therapy when indicated' or 'recognition of the problem but failure to act'."* *"Not following the guidelines should not be considered therapeutic inertia when a treatment is interrupted due to side effects or intolerance."*
Rev Med Liege 2010,**65:**232–238.	Scheen	[Inertia in clinical practice: causes, consequences, solutions.]	Belgium	Expert opinion	[Translated from french]*"Therapeutic inertia is one of the components of clinical inertia. It mainly concerns the management of chronic diseases. It may be defined as the attitude of health care providers who do not initiate or intensify therapy appropriately despite recognition of the problem."* *"It does not concern pharmacological trzatment only, but also lifestyle counseling, too ofetne neglected, especially in preventive medicine."*
J Clin Hypertens 2010,**12:**502–507.	Sutton	Why physicians do not prescribe a thiazide diuretic.	USA	Retrospective cohort study	*"Many of these reasons seem to indicate typical patterns of ''clinical inertia'' or failure to act when the BP is not controlled which contributes to overall poor BP control."* *"Many physicians do not intensify therapy when BP is close to goal."*
J Hypertens 2010;**28:**e282–283.	Van Der Niepen	Therapeutic inertia and the hawthorne effect in the management of hypertension: Results of the i-decide survey.	Belgium	Survey (poster)	*"...physician does not intend to modify the treatment, i.e. therapeutic inertia."* *"The most prevalent reasons for the no-change strategy choicewere 'the treatment is well tolerated' and'the clinical situation is acceptable'."*
J Am Soc Hypertens 2010,*4:* 244–254.	Viera	Level of blood pressure above goal and clinical inertia in a Medicaid population.	USA	Cohort study	*"The failure of clinicians to initiate or intensify antihypertensive therapy despite elevated BP levels has been termed clinical inertia One hypertension clinical action model conceptualizes clinical inertia as stemming from four domains organizational factors (e.g., sufficient support staff, access to follow-up), competing demands and prioritization (e.g., patients with several comorbidities or multiple complaints), medication related factors (e.g., number of medications, side effects), and Clinical uncertainty."*

Médecine des maladies métaboliques 2011,**5**(Suppl 2):s69–s75.	Attali	["Please don't shoot the pianist" The General practioner's point of view on clinical (or therapeutic) inertia.]	France	Expert opinion	*"Therapeutic inertia is in the heart of this debate and as far as we lack, at present, relevant qualitative tools to understand the deep reasons of the said inertia, it is necessary to be particularly suspicious with regard to the reasons sometimes too simplistic mentioned to explain this observation and with the solutions that often not take into account sick patients to offer them, under any circumstances, a better quality of care. It is a concrete situation analysis that allows us to differentiate the true inertia from the pen names inertia."*
PLoS One 2011;**6:**e24569.	Banegas	Physician perception of blood pressure control and treatment behavior in high-risk hypertensive patients: A cross-sectional study.	Spain	Cross-sectional study	*"Failure of the physician to begin or intensify treatment when the therapeutic goals are not met is a current challenge for research and action."*<br>*"'White coat'' HT and treatment non-compliance are factors often cited by physicians as contributors to therapeutic inaction."*
Aust Fam Physician 2011,**40:**24–28.	Byrnes	Why haven't I changed that? Therapeutic inertia in general practice.	Australia	Expert opinion	*"For the purposes of this article, therapeutic inertia equals clinical inertia, and is defined by Phillips et al. as the 'failure of healthcare providers to initiate or intensify therapy when indicated' and 'recognition of the problem, but failure to act'."*
Hypertension 2011,**58:**552–558.	Crowley	Treatment intensification in a hypertension telemanagement trial: clinical inertia or good clinical judgment?	USA	Randomized clinical trial	*"Clinical inertia, or provider failure to initiate or intensify hypertension therapy when indicated based on clinical guidelines."*<br>*"If implementing study conditions that counter known contributors to clinical inertia still results in frequent treatment nonintensification, an important question becomes whether all of what would typically be called clinical inertia can actually be overcome. A further question is to what extent this "refractory" clinical inertia actually represents good clinical judgment. (...) We observed that mild home BP elevations frequently did not recur even without treatment intensification. This finding suggests that clinical inertia (...) unfairly characterizes the decision not to intensify in cases when BP is near the threshold of acceptability. (...) The correct decision is whatever choice promotes the safe attainment of an acceptable BP for a given patient, and physicians must always exercise clinical judgment in determining whether to intensify therapy."*
Curr Opin Cardiol 2011,**26:**300–307.	Egan	Is blood pressure control to less than 140/less than 90 mmHg in 50% of all hypertensive patients as good as we can do in the USA: Or is this as good as it gets?	USA	Narrative review	*"Therapeutic inertia, or the failure of the healthcare provider to add or uptitrate medication when therapeutic goals are unmet."*<br>*"Therapeutic inertia often represents a patient–provider interaction, which includes the patient's reluctance to accept intensified therapy."*
Drugs Aging 2011,**28:**981–992.	Gil-Guillen	Is there a predictive profile for clinical inertia in hypertensive patients? An observational, cross-sectional, multicentre study.	Spain	Cross-sectional study	*"Inertia is a complex, mulitfactorial problem. (…)Only 4% fo the variance in clinical inertia is due to differences between physicians, the rest is the result of external factors such as patient characteristics and the clinical practice setting.(…) Many physicians in this situation not only justify clinical inertia but in fact consider it to be good clinical practice."*
JAMA 2011,**305:**1591–1592.	Giugliano	Clinical inertia as a clinical safeguard.	Italy	Commentary	*"Physicians may cite patient refusal or nonadherence as the reason for clinical inertia, it seems likely that in many instances, physician inertia is the reason for the problem."*<br>*"Clinical inertia may be a clinical safeguard for the drugintensive style of medicine fueled by the current medical literature."*<br>*"Clinical inertia may be a clinical safeguard through which physicians acknowledge the uncertainty in some current practice guidelines. An alternative explanation is that some physicians have true inertia, and thereby have unintentionally benefited patients by not prescribing new therapies or adopting more aggressive approaches for treatment of these conditions."*<br>*"Clinical inertia also may apply to the failure of physicians to stop or reduce therapy no longer needed"*<br>*"Clinical inertia is usually considered a barrier to appropriate clinician responses to asymptomatic patients"*
Médecine des maladies métaboliques 2011,**5**(Suppl 2):s62–s68.	Halimi	[Therapeutic inertia in type 2 diabetic patients: understanding without trivializing.]	France	Expert opinion	*"It often happens that this absence of modification is justified in some situations. It represents an "appropriate inertia", the result of a well- analyzed situation and a pertinent decision by the GP."*
Drugs Aging 2011,**28:**943–944.	Klein	Clinical inertia remains a problem.	USA	Editorial	*"Clinical inertia refers to the failure of healthcare providers to intervene when indicated.(...) Some contend that clinical inertia does not adequatly represent the complexity fo the patient-physician encounter. (...)Reducing this interaction to a checklist fo intervention indicated by clinical guidelines minimizes the multifaceted interplay that is the primary care visit."*<br>*"Reluctance to accept the problem of clinical inertia as a real problem is also a form of inertia."*
Hypertension 2011,**58:**544–545.	Krakoff	Guidelines, inertia, and judgment.	USA	Editorial	*"...the concept of "physician inertia" in treatment of hypertension, defined as a failure to begin or intensify treatment when the guideline says 'Do it!'"*<br>*"When physicians are systematically queried with regard to various reasons for lack of intensification,"inaction," there is remarkable consistency in their views that such decisions are not poor quality of care so that a model of inaction can be explored and analyzed with inertia being only one component for consideration."*<br>*"Before concluding that inertia is the only issue, more outcome studies are needed to categorize inaction and its consequences"*
J Clin Hypertens (Greenwich) 2011,**13:**73–80.	Nelson	Barriers to blood pressure control: a STITCH substudy.	Canada	Post-hoc analysis	*"Poor BP control linked to health care professional behavior has been most extensively studied in the context of so-called therapeutic (or clinical) inertia."*<br>*"Poor BP control is associated with clinical inertia or failure to uptitrate antihypertensive therapy."*

Médecine des maladies métaboliques 2011,**5**(Suppl 2):s57–s61.	Reach	[Strict clinical inertia: always questionable.]	France	Expert opinion	[Translated from french]*"The practitioner's behaviour is clinical inertia if and only if : (1) implicit or explicit guidelines exist; (2) the doctor is aware of the guidelines; (3) the doctor considers that the guidelines apply to the patient; (4) the doctor has the resources required to follow the guidelines; (5) the doctor does not follow the guidelines."*<br>*"If the practitioner (...) thinks that the guidelines do not apply to this particular patient, it is not clinical inertia, but appropriate inaction."*
Diabetes Metab 2012,**38**(Suppl 3):S53–58.	Avignon	Clinical inertia: viewpoints of general practitioners and diabetologists.	France	Expert opinion	*"Without a doubt, it is the result of a culture focused on figures and evaluations. Based only on the concept of absence of drug treatment intensification, clinical inertia will most likely serve as a sound box to the pharmaceutical industry to promote drug prescription."*
Diabetes Metab 2012,**38**(Suppl 3):S27–28.	Halimi	Better analyze the determinants of therapeutic inertia to overcome it.	France	Editorial	"*[Clinical inertia] can be described as insufficient surveillance and treatment, despite the fact that recommendations have been widely disseminated, are well known and can easily be put into practice."*<br>*"Their attitude may in fact reflect a careful analysis of the patient's situation, that only appears to be inertia."*<br>*"The nuances between true inertia and involuntary inertia are important(...). Nevertheless, clinical inertia and in particular therapeutic inertia in the management of chronic disorders are real, and should be identified and corrected."*
J Clin Hypertens 2013,**15:**375–379.	Desai	Prevalence of true therapeutic inertia in blood pressure control in an academic chronic kidney disease clinic.	USA	Retrospective cross-sectional study	*"Therapeutic inertia (TI) in blood pressure (BP) control has been traditionally defined as failure to initiate or intensify therapy when treatment goals are not met. The fallacy with this definition is that TI may be overestimated because it includes hypertensive patients deliberately uncontrolled."*<br>*"We conclude that the prevalence of TI in the literature overestimates the rate of true TI as it does not account for physician decision making. The current definition of TI in BP control needs to be revised, as it underestimates a provider's care to improve BP control and is misleading. The TI definition should include some mechanism to account for interventions beyond medication titration."*
J Clin Hypertens 2013,**15:**365–366.	Germino	Therapeutic inertia and measurement inertia in hypertension: a call to action.	USA	Editorial:<br>Comments on Desai et al. results and conclusions. Brings the new concept of measurement inertia	*"The authors then more narrowly define true therapeutic inertia as excluding several key populations.*<br>*- Patients whose charts clearly document an elevated BP, but a conscious decision is made to not further therapy due to concerns of overtreatment.*<br>*- Patients who refuse additional treatment.*<br>*- Patients who received additional counseling regarding lifestyle modification, with discussion documented.*<br>*- Patients whose BP is documented to have been well-controlled, with out-of-office readings that are deemed reliable despite office BP readings that may be elevated.*<br>*These are all reasonable modifications to the term therapeutic inertia and add greater precision to the term."*<br>*"The lack of consistent evidence that repeat measurements were performed during the visits of these patients with hypertension and CKD, seen by nephrologists in an academic center, is disconcerting and represents a different type of inertia—that of measurement inertia."*
Int J Clin Pract 2013,**67:**97–98.	Rodrigo	Therapeutic momentum: a concept opposite to therapeutic inertia.	Sri-Lanka	Letter:<br>Based on a study of the prescription patterns fo antiplatelet drugs as secondary prohylaxis	*"The reluctance to step down or withdraw therapy when further prescription is not needed or not supported by evidence. We have termed it 'therapeutic momentum'."*<br>*"This concept is the opposite to the concept of therapeutic inertia, but can be calculated using a similar equation (expected rate of reduction or stoppage of medication – observed rate of reduction or stoppage of medication)."*
